# Supplementary material for: Generated Randomly and Selected Functionally? The Nature of Enterovirus Recombination
Source: Viruses. 2022 Apr 28;14(5):916. doi: 10.3390/v14050916 (PMC9144335; doi:10.3390/v14050916)
Supplement: Supplementary file 1 [file viruses-14-00916-s001.zip › viruses-1661232-supplementary.pdf]

viruses

MDPI

Supplementary

Generated Randomly and Selected Functionally? The Nature of Enterovirus

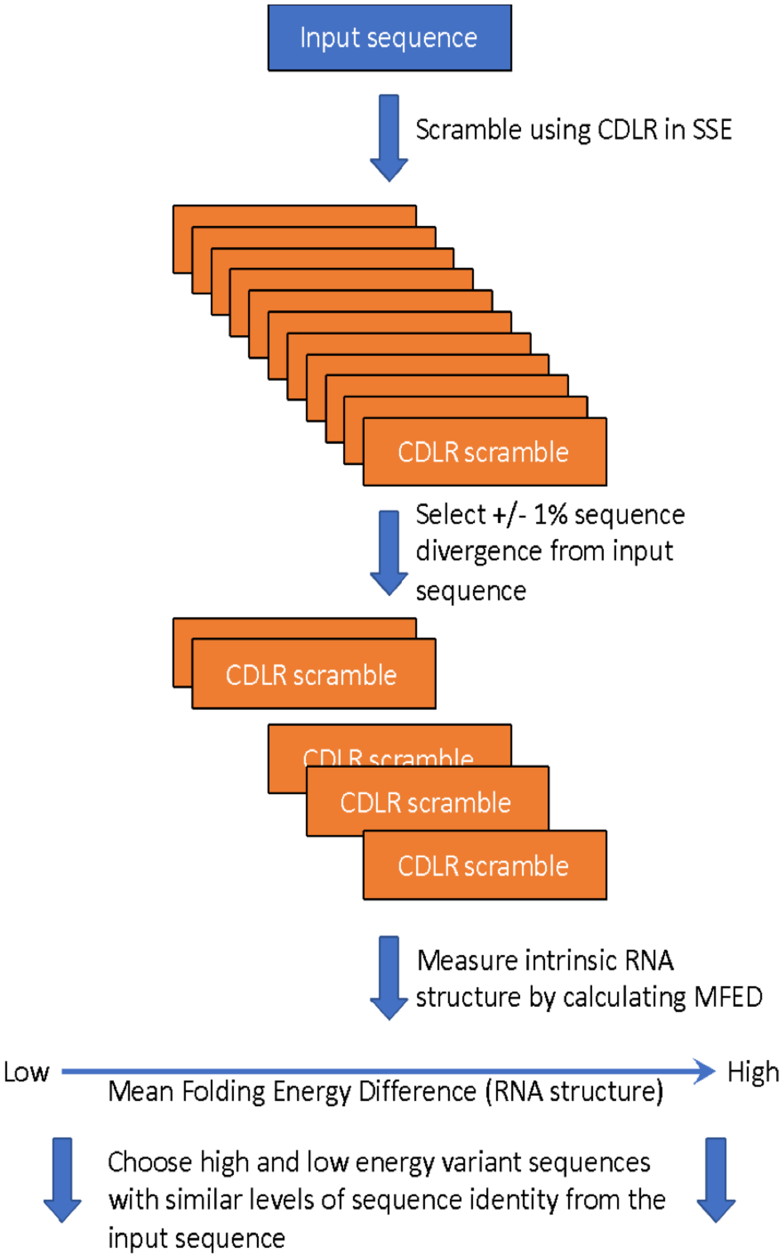

Figure S1. Design flowchart for RNA sequences with high or low levels of RNA structure. The input sequences were scrambled using the CDLR algorithm within the SSE package (reference 25). This method scrambles codon order, but maintains the open reading frame, the encoded polyprotein, the dinucleotide and codon frequencies. Scrambled sequences within +/-1% sequence divergence from the input sequence were selected. Each was analysed and ordered for inherent RNA structure by calculating the mean folding energy difference (MFED) between the CDLR scrambled sequences and 99 sequence-order randomised variants (see reference 34). Representative high and low structured variants, both exhibiting similar sequence identity to the native input poliovirus sequence, were selected from the 10 sequences with the highest or lowest level of intrinsic structure respectively.

```
CCAGGGUCUGGAUGC – donor

AGCCACGACGGCGAU – acceptor

AGCCACGACGGCUGC – precise, unambiguous

AGCCACGACGGAUGC – precise, ambiguous

AGCCACGAAUGC – imprecise, unambiguous

AGCCACGACGGUGGAUGC – imprecise, unambiguous

AGCCACGGAUGC – imprecise, ambiguous

AGCCACGACGGGAUGC – imprecise, ambiguous
```

Figure S2. Recombination junction definition. Donor and recipient sequences are shown in red and blue respectively. Precise junctions have no insertions or deletions with respect to the length of the parental sequences. Imprecise junctions contain insertions or have sequences deleted. The position of unambiguous junctions can be defined by the position of the join in the donor and acceptor sequences. Ambiguous junctions, whether in precise or imprecise junctions, cannot be exactly defined due to limited sequence identity between donor and acceptor at the junction, and are highlighted in black underlined text. The two thin vertical lines refer to start and end of the precise recombinants, to visually aid reading the figure. .

```
PV1     TTCGTTGGCCCAACGTTCCAGTACATGGAGGCTAATAACTATTACCCAGCTAGGTACCAGTCCCATATGCTCATTGGCCATGGATTCGCATCTCCAGGGGATTGTGGTGGCATACTCAGA  3718
PV1^L   TTCGTTGGCCCCACGTTTCAGTATATGGAAGCTAATAACTATTACCCAGCAAGATACCAGTCTCACATGCTCATAGGCCATGGGTTTGCCTCCCCCGGGGACTGTGGGGGATACTTAGG  3718
PV1^H   TTCGTTGGCCCAACATTCCAGTACATGGAAGCCAATAACTATTACCCAGCTAGATACCAGTCTCATATGCTTATCGGTCATGGATTCGCGTCTCCAGGTGACTGTGGTGGCATTCTTAGA  3718
PV1^3   TTTGTGGGACCCACCTTCCAATACATGGAGGCTAATAACTACTACCCAGCTAGATACCAATCCCACATGTTAATCGGGCACGGCTTTGCCTCACCAGGTGACTGTGGTGGTATCCTTAGG  3718
PV3     TTTGTGGGACCCACCTTCCAATACATGGAGGCTAATGACTACTACCCAGCTAGATACCAATCCCACATGTTAATCGGGCACGGCTTTGCCTCACCAGGTGACTGTGGTGGTATCCTTAGG  3718
PV3^L   TTTGTTGGGCCCACCTTCCAATATATGGAGGCAAATGACTACTACCCTGCCAGATACCAATCTCATATGTTAATCGGACACGGCTTTGCTTCTCCAGGTGACTGTGGTGGAATTCTAAGA  3718
PV3^H   TTCGTGGGGCCAACATTTCAATACATGGAAGCCAATGACTATTACCCAGCTAGGTACCAATCCCACATGTTGATCGGACACGGCTTTGCATCTCCTGGCGACTGTGGTGGTATCCTCAGA  3718
PV3^1   TTCGTTGGCCCAACGTTCCAGTACATGGAGGCTAATGACTATTACCCAGCTAGGTACCAGTCCCATATGCTCATTGGCCATGGATTCGCATCTCCAGGGGATTGTGGTGGCATACTCAGA  3718
        ** ** ** ** ** ** ** ** ***** ** *** **** ***** ** ** ***** ** ** *** * ** ** ** ** ** ** ** ** ** ** ***** ** ** ** **

PV1     TGTCACCACGGGGTGATAGGGATCATTACTGCTGGTGGCGAAGGGTTGGTTGCATTTTCAGACATTAGAGACTTGTATGCCTACGAAGAAGAAGCCATGGAACAAGGCATCACCAATTAC  3838
PV1^L   TGTCACCACGGAGTGATTGGCATTATTACAGCAGGTGGCGAAGGATTGGTTGCATTTTCAGACATCAGGGATTTGTATGCCTATGAAGAAGAAGCCATGGAGCAGGGTATAACAAATTAC  3838
PV1^H   TGCCACCACGGGGTTATAGGGATCATCACAGCTGGTGGGGAGGGGTTGGTTGCCTTTTCCGACATTAGGGATTTGTATGCCTACGAAGAAGAAGCTATGGAGCAAGGTATTACCAATTAC  3838
PV1^3   TGTCACCATGGCGTCATCGGAATCATTACAGCTGGTGGAGAGGGATTAGTCGCATTCTCTGACATAAGGGACTTGTATGCTTACGAGGAAGAGGCCATGGAGCAGGGCATTACAAACTAT  3838
PV3     TGTCAACATGGCGTCATCGGAATCGTGACAGCTGGTGGAGAGGGATTAGTCGCATTCTCTGACATAAGGGACTTGTATGCTTACGAGGAAGAGGCCATGGAGCAGGGCATTTCAAACTAT  3838
PV3^L   TGTCAGCACGGTGTAATCGGTATAGTGACTGCAGGGGGTGAGGGGTTAGTTGCGTTTTCAGACATAAGGGACTTGTATGCCTACGAGGAAGAGGCGATGGAGCAGGGCATTTCCAACTAC  3838
PV3^H   TGTCAGCACGGAGTTATCGGAATAGTTACAGCAGGTGGTGAAGGGTTAGTCGCCTTCTCTGACATTAGGGACTTGTATGCCTATGAGGAGGAGGCGATGGAGCAGGGTATTTCCAACTAT  3838
PV3^1   TGTCAACACGGGGTGATAGGGATCGTGACTGCTGGTGGCGAAGGGTTGGTTGCATTTTCAGACATTAGAGACTTGTATGCCTACGAAGAAGAAGCCATGGAACAAGGCATCTCAAATTAC  3838
        ** ** ** ** ** ** ** **  * ** ** ** ** ** ** ** ** ** ** ** ***** ** ** ******** ** ** ** ** ** ***** ** ** **  * ** **

PV1     ATAGAGTCACTTGGGGCCGCATTTGGAAGTGGATTTACTCAGCAGATTAGCGACAAAATAACAGAGTTGACCAATATGGTGACCAGTACCATCACTGAAAAGCTACTTAAGAACTTGATC  3838
PV1^L   ATAGAGTCACTCGGTGCTGCATTTGGGAGCGGGTTCACTCAACAGATTAGTGACAAAATAACAGAGTTGACTAACATGGTCACTAGCACCATCACTGAAAAGCTACTTAAGAACTTGATC  3838
PV1^H   ATAGAGTCCCTAGGAGCTGCATTTGGGAGCGGGTTCACACAGCAGATCAGTGACAAAATCACTGAGCTGACCAATATGGTGACCAGCACCATAACAGAGAAACTGTTGAAAAACTTGATT  3838
PV1^3   ATTGAGTCACTCGGTGCTGCGTTCGGTAGTGGGTTCACTCAGCAAATAAGCGATAAGATAACAGAACTAACCAACATGGTGACCAGCACGATTACAGAGAAGCTACTTAAAAACCTAATC  3838
PV3     ATTGAGTCACTCGGTGCTGCGTTCGGTAGTGGGTTCACTCAGCAAATAGGGGATAAGATATCAGAACTAACCAGCATGGTGACCAGCACGATTACAGAGAAGCTACTTAAAAACCTAATC  3838
PV3^L   ATCGAGTCACTGGGAGCAGCATTCGGTAGTGGGTTCACCCAACAAATAGGCGACAAAATATCAGAACTTACTAGCATGGTTACCAGCACTATCACAGAGAAGCTACTGAAAAACCTGATC  3838
PV3^H   ATTGAGTCTCTCGGTGCAGCTTTTGGCAGTGGGTTCACTCAACAAATCGGGGATAAGATTTCTGAGCTAACTAGCATGGTAACCAGCACCATTACAGAAAAACTGCTGAAAAACCTAATC  3838
PV3^1   ATAGAGTCACTTGGGGCCGCATTTGGAAGTGGATTTACTCAGCAGATTGGGGACAAAATATCAGAGTTGACCAGCATGGTGACCAGTACCATCACTGAAAAGCTACTTAAGAACTTGATC  3838
        ** ***** ** ** ** ** ** ** ** ** ** ** ** ** **  * ** ** **  * **  * ** *  ***** ** ** ** ** ** ** ** **  * ** *** * **

PV1     AAGATCATATCCTCACTAGTTATTATAACTAGGAACTATGAAGACACCACAACAGTGCTCGCTACCCTGGCCCTTCTTGGGTGTGAT    4045
PV1^L   AAGATTATATCATCACTCGTGATCATTACCAGAAACTACGAAGATACCACTACTGTGCTAGCCACCCTGGCCCTCCTTGGATGTGAT    4045
PV1^H   AAGATCATATCATCCCTGGTTATAATCACCAGAAACTATGAAGACACTACAACAGTACTTGCTACCCTTGCACTACTTGGGTGCGAT    4045
PV1^3   AAAATTATTTCATCTCTGGTGATTATCACTAGAAATTACGAAGATACCACCACAGTGCTCGCCACTCTAGCTCTTCTTGGGTGTGAT    4045
PV3     AAAATTATTTCATCTCTGGTGATTATCACTAGAAATTACGAAGATACCACCACAGTGCTCGCCACTCTAGCTCTTCTTGGGTGTGAT    4045
PV3^L   AAGATCATCTCATCACTGGTTATTATTACCAGAAATTATGAAGATACCACTACTGTCCTCGCCACTCTCGCTCTGCTCGGGTGTGAT    4045
PV3^H   AAGATAATATCGTCACTGGTGATTATCACCAGAAACTACGAGGATACCACCACAGTACTCGCTACACTAGCTCTCCTTGGATGTGAT    4045
PV3^1   AAGATCATATCCTCACTAGTTATTATAACTAGGAACTATGAAGACACCACAACAGTGCTCGCTACCCTGGCCCTTCTTGGGTGTGAT    4045
        ** ** ** ** ** ** ** ** ** ** ** ** ** ** ** ** ** ** ** ** ** ** ** ** ** ** ** ** ***
```

Figure S3. Sequence alignments of modified templates. The modified Cluster 2 sequences were aligned against parental PV1 and PV3 sequences using Clustal Omega.

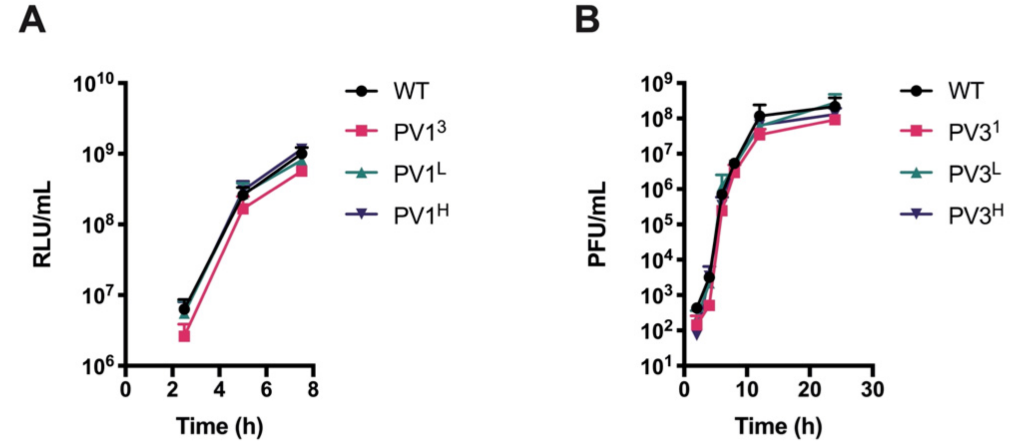

Figure S4. CRE-REP modified genome showed comparable characteristics to WT CRE-REP geneoms. (A) Replication kinetics for donor RNAs. HeLa cells were transfected with 200 ng RNA/well in 24-well plates and luciferase activity measured over an 8 h time course. Error bars represent the standard deviation of three experiments with samples assayed in triplicate. (B) Replication kinetics for acceptor RNAs. HeLa cells were infected with virus stock at MOI of 5 and virus-containing supernatants harvested over 24 h. Virus titres were determined by plaque assay on HeLa cells. Error bars represent the standard deviation of three experiments.

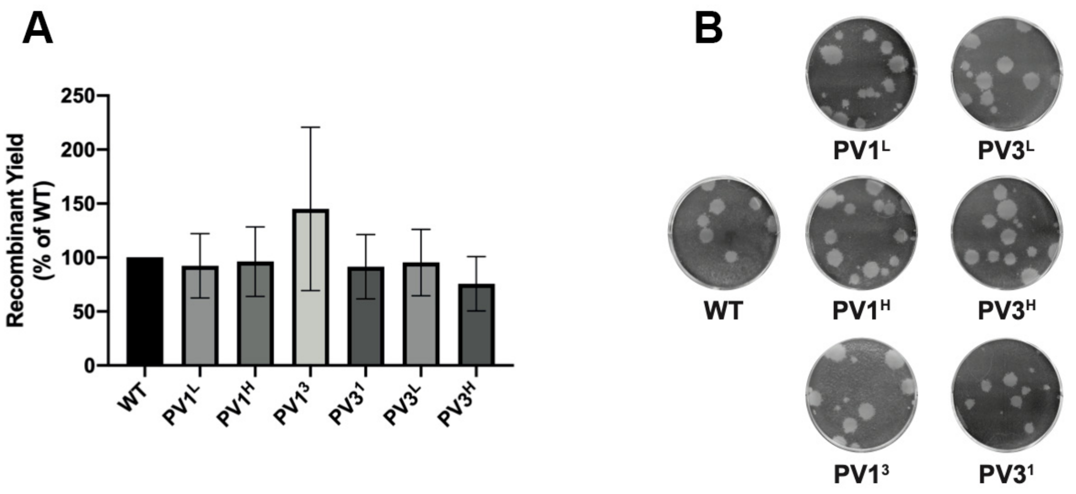

Figure S5. Quantification and characterisation of donor and acceptor RNAs in modified CRE-REP assays. (A) Recombinant yield from modified CRE-REP assays. RNAs were co-transfected into L929 cells in equimolar amounts and virus-containing supernatants were harvested 30 h post-transfection. Recombinant virus yields were determined by plaque assay on HeLa cells and expressed as a percentage of the WT assay. Error bars represent standard error of the mean of three co-transfection experiments. All modified assays were not significantly different to WT, as determined by unpaired t-test. (B) Plaque morphology comparison between recombinant viruses obtained from each of the CRE-REP assays.

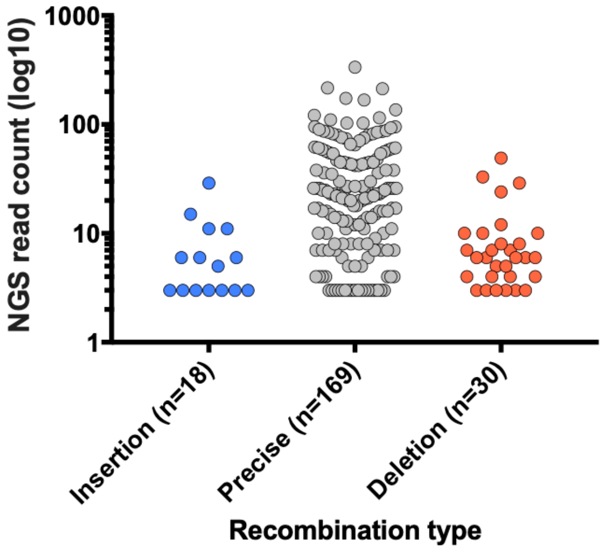

Figure S6. Sequencing analysis of the co-infection technical replicate sample. The number of precise and imprecise (insertion and deletion) recombinants was plotted against the number of NGS reads (left panel). Each dot represents a unique recombinant.

Table S1. Percentage sequence identity of structure modified sequences.

	**PV1**	**PV1L**	**PV1H**	**PV3**	**PV3L**	**PV3H**
**PV1**	-	*85.46%*	*85.46%*	*77.63%*	*78.52%*	*77.40%*
**PV3**	*77.63%*	*78.75%*	*76.96%*	-	*85.46%*	*85.46%*
ΔG (kcal/mol)	*-124.90*	*-112.30*	*-149.90*	*-140.00*	*-126.20*	*-149.80*

Table S2. Recombination junctions isolated by PCR cloning and sequenced by Sanger-sequencing. (Del = Deletion, Prec = Precise, Ins = Insertion).

**PV1**	**PV1L**	**PV1H**
Del	4500	3848
Del	4339	3372
Prec	4156	4156
Prec	4020	4020
Prec	3777	3777
Prec	3600	3600
Ins	4045	4100
